# A Suitable HPLC-MS/MS Methodology for the Detection of Oxytetracycline, Enrofloxacin, and Sulfachloropyridazine Residues in Lettuce Plants

**DOI:** 10.3390/foods13010153

**Published:** 2024-01-02

**Authors:** Karina Yévenes, María José Ibáñez, Ekaterina Pokrant, Andrés Flores, Matías Maturana, Aldo Maddaleno, Javiera Cornejo

**Affiliations:** 1 Department of Preventive Animal Medicine, Faculty of Veterinary and Animal Sciences, University of Chile, Santiago 8820808, Chile; kyevenes@ug.uchile.cl (K.Y.); maria.ibanez.d@ug.uchile.cl (M.J.I.); katiavalerievna@uchile.cl (E.P.); 2 Doctorate Program of Forestry, Agricultural and Veterinary Sciences (DCSAV), Southern Campus, University of Chile, Santa Rosa 11315, La Pintana, Santiago 8820808, Chile; 3 Laboratory of Veterinary Pharmacology (FARMAVET), Faculty of Veterinary and Animal Sciences, University of Chile, Santiago 8820808, Chile; andres.flores@veterinaria.uchile.cl (A.F.); matias.maturana.medina@gmail.com (M.M.); amaddaleno@veterinaria.uchile.cl (A.M.)

**Keywords:** oxytetracycline, enrofloxacin, sulfachloropyridazine, antimicrobial residues, single validation, HPLC/MS-MS method, lettuce

## Abstract

Oxytetracycline (OTC), enrofloxacin (EFX), and sulfachloropyridazine (SCP) are critically important antimicrobials (AMs) in both human and veterinary medicine, where they are widely used in farm animals. Lettuce has become a matrix of choice for studying the presence of residues of these AMs in plants, as the concentrations of residues detected in lettuce can range from ng to mg. While several analytical methodologies have been developed for the purpose of detecting AMs in lettuce, these currently do not detect both the parent compound and its active metabolites or epimers, such as in the case of ciprofloxacin (CFX) and 4-epi-oxitetracycline (4-epi-OTC), which also pose a risk to public health and the environment due to their AM activity. In light of this situation, this work proposes an analytical method that was developed specifically to allow for the detection of OTC, 4-epi-OTC, EFX, CFX, and SCP in a lettuce matrix. This method uses acetonitrile, methanol, 0.5% formic acid, and McIlvaine-EDTA buffer as extraction solvents, and dispersive solid-phase extraction (dSPE) for the clean-up. The analytes were detected using a liquid chromatography technique coupled to mass spectrometry (HPLC-MS/MS). Parameters such as the specificity, linearity, recovery, precision, limit of detection, and limit (LOD) of quantification (LOQ) were calculated according to the recommendations established in the European Union decision 2021/808/EC and VICH GL2: Validation of analytical procedures. The LOQ for the analytes OTC, 4-epi-OTC, CFX, and SCP was 1 μg·kg^−1^, whereas for EFX, it was 5 μg·kg^−1^ dry weight. All calibration curves showed a coefficient of determination (R^2^) of >0.99. The recovery levels ranged from 93.0 to 110.5% and the precision met the acceptance criteria, with a coefficient of variation of ≤14.02%. Therefore, this methodology allows for the precise and reliable detection and quantification of these analytes. The analysis of commercial samples confirmed the suitability of this method.

## 1. Introduction

Antimicrobials (AMs) can enter agricultural systems through several routes. One of them is via the fertilization of soils using manure [1], which, along with urine, is a vehicle for the excretion of residues of all the AMs administered to farm animals (10% to 90% of the administered doses). Additionally, unused or expired medications are commonly disposed of in water that is later poured onto soil [2]. Once these AMs are incorporated into the soil, they decompose, resulting in low AM concentrations. These pose a serious problem, as bacterial exposure to sub-lethal concentrations tends to favor the adaptation and proliferation of bacteria that show resistance to multiple AMs [3].

But soil may not be the final destination of AMs, as these can be transported away in run-off waters, or via lixiviation into surface and/or deep groundwater, or even be absorbed by plants and stored into their tissues [4]. When these run-off waters reach plant roots, AMs accumulate there before entering the plant’s vascular system through the apoplastic, symplastic, or transmembrane pathway. The presence of AM drugs in plants can have several impacts. Negative effects on plant growth and yield are described due to the phytotoxic effects [5,6]. Likewise, AM drug residues can lead to the development of several adverse effects on human health, such as hypersensitivity reactions, hepatotoxicity, carcinogenic and mutagenic effects, teratogenicity, reproductive disorders, and disruption of the normal intestinal microbial flora [7].

However, the greatest risk associated with the presence of AM residues is the continued selective pressure on bacteria in humans and the environment, selecting for microorganisms with AM resistance genes (ARGs), which can persist in commensal bacteria and be transferred to pathogenic microorganisms [8,9].

This background is relevant if we consider that AM resistance has been prioritized by the World Health Organization (WHO) as one of the top 10 global public health threats facing humanity, potentially responsible for as many as 10 million deaths annually by 2050 [10].

Lettuce is one of the main plants wherein AM residues are routinely studied. This is mostly due to their availability, as worldwide production is greater than 27 million tons [11], but also because they are consumed fresh, which avoids any AM degradation that could result from cooking processes [12]. In fact, several in vivo studies have provided evidence regarding the accumulation of AM residues in lettuce [13,14,15,16,17]. For instance, a field study found concentrations ranging between 4.4 and 33.6 ng·g^−1^ fresh weight of different classes of AMs in lettuce sourced from farms and markets in the city of Kumas, Ghana [18]. Likewise, Yu et al. [19] detected tetracycline residues in lettuce samples purchased at a market in Beijing, at concentrations of 8.84 μg·kg^−1^ of dry matter.

Tetracyclines, quinolones, and sulfonamides are some of the AM classes most frequently detected in crops [4], probably because they are among the most commonly used AMs in the animal industry [20]. In particular, oxytetracycline (OTC), enrofloxacin (EFX), and sulfachloropyridazine (SCP) are the main AM drugs currently used in Chile [21], as these AMs show molecular characteristics at the pharmacokinetic level, as well as regarding their behavior within the environment, making them especially desirable [3].

Properly defining a general mapping of AM contamination in the environment requires the development of suitable analytical methods that allow us to quantify AMs in complex biological matrices, such as food. To this end, the confirmatory technique of choice when determining AMs in various samples is liquid chromatography coupled to mass spectrometry (HPLC-MS/MS). Using this highly sensitive and selective method allows for separating the compounds using HPLC and then directly identifying them using MS/MS based on their masses [22].

When assessing the presence of AM residues in different kinds of samples, it is important to determine not only the parent compound but also its active metabolites or epimers. For instance, after administering EFX to broiler chickens, 74% of the drug is excreted as the original molecule, but approximately 25% of it is excreted as its main active metabolite, ciprofloxacin (CFX) [23]. Meanwhile, whenever OTC is exposed to a pH between 2 and 6, the molecule can reversibly epimerize in the C-4 position, becoming 4-epi-oxytetracycline (4-epi-OTC) [24]. Consequently, these molecules are also used as residue markers and must always be assessed along with their parent compound. For the particular case of SCP, its metabolites do not have AM activity; thus, they are not deemed as marker metabolites that need to be accounted for [25].

Currently, several methodologies allow for the detection of AMs in lettuce [12,13,15,18,26,27]. However, we are not aware of any studies that involve the detection of both the parent molecules and its main active metabolites and epimers, such as CFX and 4-epi-OTC. As these molecules also show AM activity, they pose a risk to public health and the environment. Therefore, this work intended to implement and validate an analytical methodology that allows for the detection of residues of OTC, 4-epi-OTC, EFX, CFX, and SCP in lettuce samples, and to verify this methodology through the detection of AM drug residues in commercially available lettuce.

## 2. Materials and Methods

### 2.1. Sample Collection

To ensure that lettuce samples were free of AM residues, commercial seeds of butter lettuce, *Lactusa Sativa*, var. Capitata (ANASAC^®^), were sowed on land provided by the Laboratory of Soil and Water Chemistry of the Faculty of Agronomic Sciences of the University of Chile. The soil was typical of central Chile. These plants were then harvested on day 55 post-germination and subsequently chopped and freeze-dried in a LABCONCO© Free Zone 2.5 device (Kansas City, MO, USA) before finally storing them in plastic bags at –20 °C.

### 2.2. Standards, Reagents, and Chemicals

The standards for oxytetracycline (OTC), 4-epi-oxytetracycline (4-epi-OTC), enrofloxacin (EFX), ciprofloxacin (CFX), and sulfachloropyridazine (SCP) used in this work were of certified purity (>99.7%) and sourced from Sigma Aldrich, Inc. (Merck KGaA). As for the internal standards (ISt) of tetracycline-D6 (TC-D_6_), enrofloxacin-D5 (EFX-D_5_), and sulfamethazine 13C6 (SMZ-13C_6_), they also were of certified purity (>99.7%) and sourced from Toronto Research Chemicals (Toronto, ON, Canada). 

The stock solutions of OTC, 4-epi-OTC, EFX, CFX, and SCP were prepared in methanol at a concentration of 1000 mg·L^−1^ and then stored at −80 °C. The intermediate (working) solutions were prepared by diluting the stock solution in methanol down to a concentration of 1000 µg·L^−1^ and then also stored at −80 °C.

The McIlvaine-EDTA buffer (0.1 M, pH 4.0) used to extract the analytes was prepared by mixing 500 mL of solution *A* with 312.5 mL of solution *B* and then adding 3.72 g of EDTA. Solution *A* was prepared by diluting 14.2 g of disodium hydrogen phosphate dihydrate in 500 mL of water, whereas solution *B* was prepared by diluting 10.5 g of citric acid monohydrate in 500 mL of water. 

Formic acid solutions of 0.1% and 0.5% *v/v* concentration were also used in this work. These were prepared by diluting 100 or 500 µL of formic acid, respectively, in 100 mL of water. All other reagents, such as water, formic acid, methanol, and acetonitrile, were of analytical grade and sourced from either Fisher (Thermo Fisher Scientific, Waltham, MA, USA), Merck (Merck, Darmstadt, Germany), or a similar company.

### 2.3. Pre-Treatment and Extraction Proceedure of Antimicrobial Residues from Lettuce Samples

The pre-treatment consisted of cutting fresh lettuce leaves into small pieces and placing them in glass containers to freeze them for 12 h at −20 °C. The freeze-drying process was performed in a LABCONCO^©^ Free Zone 2.5 (Kansas City, MO, USA) and consisted in a primary drying (ice sublimation) and a secondary drying (desorption). Then, once the dried sample was obtained, it was mixed and shredded in a mortar to obtain the freeze-dried lettuce. 

**Figure 1:**
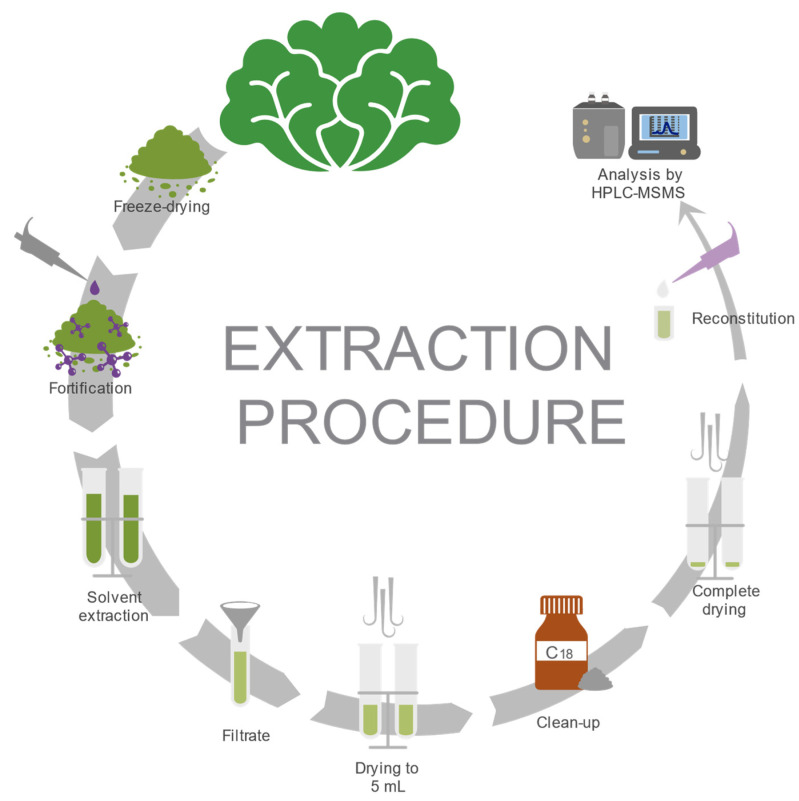
Solid–liquid extraction of oxytetracycline, 4-epi-oxytetracycline, enrofloxacin, ciprofloxacin, and sulfachloropyridazine from lettuce.

The analysis began by collecting 0.2 g of freeze-dried lettuce (equivalent to 3 g of lettuce) in a 50 mL polypropylene tube and then fortifying it with working solutions. Afterward, 5 mL of a mixture of acetonitrile, methanol, 0.5% formic acid, and EDTA buffer in a ratio of 40:10:20:30 (*v*/*v*/*v*/*v*) was added to the sample. The vials were then agitated for 5 min, sonicated for 15 min, and centrifuged for 10 min at 2700× *g* in a Hettich^®^ ROTOFIX 32A centrifuge (Hettich Lab Technology, Beverly, MA, USA). Then, the supernatant was sieved through a Whatman™ glass fiber filter of grade GF/A 1.6 µm (MERCK, Darmstadt, Germany) while being transferred into another 50 mL polypropylene tube. The whole extraction process was repeated twice. Both supernatants were then mixed and reduced down to 5 mL under a nitrogen flow in a water bath at a temperature of 45 ± 5 °C. Each sample was cleaned up by means of dispersive solid-phase extraction (dSPE) using 1 g of bulk C_18_, shaken for 2 min, and centrifuged at 2700× *g* for 10 min. The resulting supernatant was poured into a glass tube and dried out under a nitrogen flow in a water bath at a temperature of 45 ± 5 °C. Next, each sample was reconstituted up to a 1.5 mL solution using a solvent mix of acetonitrile and 0.1% formic acid (at a ratio of 20:80 *v*/*v*) and poured into a 1.5 mL Eppendorf^®^ tube that was centrifuged at 17,136× *g* for 10 min at 0 °C using a VWR^®^ 2417R device (Avantor, Radnor, PA, USA). Finally, the supernatant was collected using a 3 mL syringe and sieved through a 33 mm Millex^®^ filter with 0.22 µm pore size (Merck KGaA, Burlington, MA, USA) into a glass vial, ready to be run through an HPLC MS/MS device (Figure 1).

### 2.4. Instrumental Analysis

The samples were analyzed using an Agilent^®^ Series 1290 liquid chromatograph (Agilent Technologies, Santa Clara, CA, USA) coupled to an ABSCIEX^®^ API 5500 mass spectrometer equipped with an electrospray ionization (ESI) source (SCIEX, Framingham, MA, USA) and a Sunfire^TM^ C_18_ chromatography column (Waters Corp., Milford, MA, USA) of dimensions 3.5 μm and 150 × 2.1 mm. The analytes were chromatographically separated using a mobile phase gradient of 0.1% formic acid in water (Phase A) and 0.1% formic acid in methanol (Phase B). The flow rate was set at 0.2 mL·min^−1^, for an injection volume of 20 µL and a column temperature of 35 ± 1 °C. Table 1 shows the elution gradient.

The retention times for OTC and 4-epi-OTC were 10.18 and 7.60 min, respectively. For EFX and CFX, these were 9.83 and 9.47 min, respectively. For SCP, it was 12.98 min. Meanwhile, the average retention times for EFX-D_5_, SMZ-13C_6_, and TC-D_6_ were 9.77, 11.37, and 9.35 min, respectively. 

The software Analyst^®^ version 1.6.2 (SCIEX, Framingham, MA, USA) was used for the chromatographic integration of samples.

For MS/MS analysis, positive multiple reaction monitoring mode (+MRM) was applied with the following parameters: curtain gas 30 psi, collision gas 10 psi, source temperature 550 °C, electro-spray voltage 3500 V, ion source gas one 60 psi, and ion source gas two 80 psi. 

The criterion for identifying the different AMs and their active metabolites was the detection of the masses of the precursor and fragmented ions (Table 2). All samples were processed and analyzed in the Laboratory of Veterinary Pharmacology (FARMAVET, by its Spanish acronym) in the Faculty of Veterinary and Animal Sciences at the University of Chile, as this laboratory is accredited under ISO/IEC number 17025:2017 [28]. 

### 2.5. In-House Validation of the Analytical Method

To develop the in-house validation of the analytical method, an internal protocol specially designed for this study was implemented following the recommendations provided by the European Commission Decision 2021/808/EC [29] and VICH GL2: Validation of analytical procedures: methodology [30]. This single validation assesses parameters such as the specificity, limit of detection (LOD), limit of quantification (LOQ), recovery, precision (assessed by both repeatability and intralaboratory reproducibility), and linearity of the calibration curve.

To determine the LOD and the LOQ, the signal-to-noise ratio of the analyte was expected to fall within the range of ≥3:1 and ≥10:1, respectively. Recovery was assessed as the amount of analyte recovered in the final stage of the analytical process divided by the amount of analyte in the original sample, expressed as a percentage. The range of expected values was 80–120% for six calibration curves fortified at three different concentration levels (1, 5, and 15 µg·kg^−1^ dry weight (dw) for the analytes OTC, 4-epi-OTC, and SCP, and 5, 15, and 30 µg·kg^−1^ dw for the analytes EFX and CFX).

Regarding precision, this parameter was assessed by analyzing the repeatability and the intralaboratory reproducibility. In the case of repeatability, six calibration curves were fortified and processed at three different concentration levels on the same day (1, 5, and 15 µg·kg^−1^ dw for the analytes OTC, 4-epi-OTC, and SCP, and 5, 15, and 30 µg·kg^−1^ dw for the analytes EFX and CFX). Meanwhile, intralaboratory reproducibility was measured by using six calibration curves, fortified at the same concentration levels as for the repeatability assessment, but these were analyzed on different days and by different analysts. The CV of reproducibility must be lower than the CV of repeatability. The specificity of the method was calculated by analyzing 20 blank samples.

Finally, the linearity of the calibration curve was assessed using three calibration curves fortified at 5, 15, 30, 60, and 120 µg·kg^−1^ dw for the analytes EFX and CFX and at 1, 5, 15, 30, and 60 µg·kg^−1^ dw for the analytes OTC, 4-epi-OTC, and SCP. The coefficient of determination (R^2^) and the slope of the regression line for each of the curves were determined. 

### 2.6. Detection of Antimicrobial Drug Residues in Commercially Available Lettuce 

The samples were collected following a non-probabilistic convenience sampling, as described by Otzen and Manterola [31]. To this end, lettuce samples were collected from Lo Valledor and La Vega Central, which are the main wholesale markets in the Metropolitan Region of Chile [32]. Three producers were chosen at each market, and 4 lettuces were selected per producer at each of these locations, resulting in a total of 24 samples. 

These samples were then chopped and stored frozen at −20 °C, awaiting further processing to extract and detect the analytes via chromatographic techniques. The results were then quantified by means of regression analysis of the calibration curves calculated previously in this matrix. The calculated R^2^ was expected to be greater than 0.95.

The confirmation criteria to determine positive samples were the analyte retention time (≤2.5%), the relative retention time (≤2.5%), the signal to noise (three times), and the ratio (according to relative intensity).

## 3. Results 

### 3.1. Implementation and Optimization of the Analytical Methodology

The extraction methodology was designed on the basis of the method previously published by Albero et al. [12] but incorporated the addition of EDTA buffer as a solvent and 1.6 µm Whatman^TM^ glass microfiber paper filters of GF/A grade (MERCK, Darmstadt, Germany) during the clean-up process. The reason for adding the EDTA buffer was that it would act as a cation chelator, and the paper filter was used to improve the cleanliness of the samples. Both modifications reduced any interference that could impact the chromatographic analysis. 

### 3.2. In-House Validation of the Analytical Methodology on Lettuce Samples

The analytical methodology developed for this research was validated by calculating the parameters detailed below.

#### 3.2.1. Specificity

**Figure 2:**
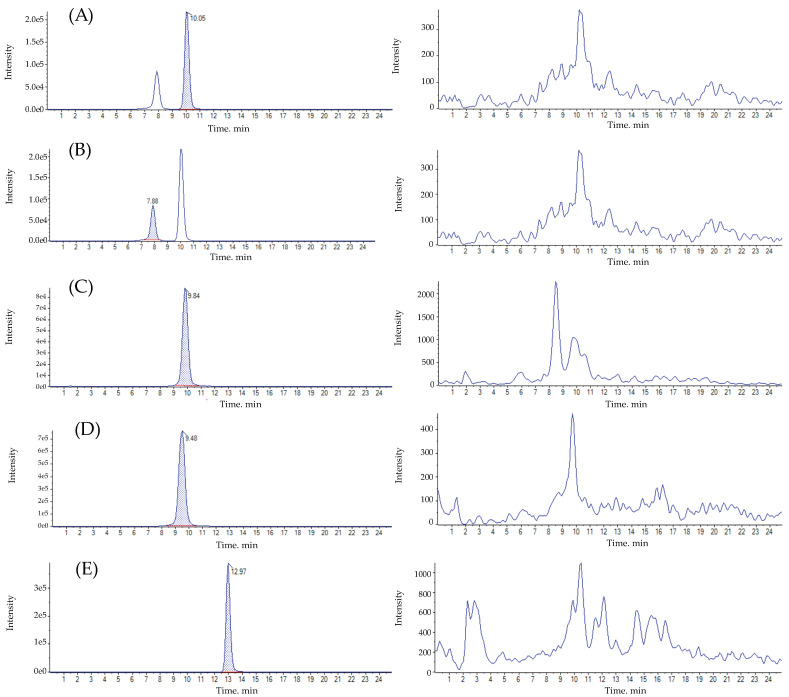
On the left, chromatograms of (**A**) OTC, (**B**) 4-epi-OTC, (**C**) EFX, (**D**) CFX, and (**E**) SCP for an internal standard solution (50 ng·mL^−1^ of either drug). On the right, the respective blank lettuce samples.

A total of 20 samples deemed to be free from residues of the examined AMs were analyzed to rule out the existence of any interference on the retention time for each analyte. The results from these analyses showed no interference signals on the retention time (Figure 2).

#### 3.2.2. Detection Range

The LOD values for OTC, 4-epi-OTC, CFX, and EFX were established based on the signal-to-noise ratio approach. Thus, for OTC, 4-epi-OTC, CFX, and EFX, the LOD was set at 0.8 µg·kg^−1^ dw, while for EFX, it was 4.5 µg·kg^−1^ dw, when the signal-to-noise ratio of the fortified matrix was higher than 3:1 for each analyte. The LOQ values for OTC, 4-epi-OTC, CFX, and EFX were set at the concentration at which a signal-to-noise ratio higher than 10:1 was observed. Thus, the LOQ value set for OTC, 4-epi-OTC, CFX, and EFX was 1 µg·kg^−1^ dw, while for EFX, it was 5 µg·kg^−1^ dw.

#### 3.2.3. Linearity of Calibration Curves

Five concentration levels (5, 15, 30, 60, and 120 µg·kg^−1^ dw for the analytes EFX and CFX and 1, 5, 15, 30, and 60 µg·kg^−1^ dw for the analytes OTC, 4-epi-OTC, and SCP) were used to prepare the calibration curves. The validation of this parameter was accepted because the determination coefficients (R^2^) were greater than 0.99 (Table 3) for all analytes, and their CV was less than 25% as well. Moreover, the slope analysis indicated that lineal equations were slightly variable, less than 20%. Consequently, the observed values showed no significant variations on linearity that could affect the robustness of the analytical results.

#### 3.2.4. Recovery and Precision

Recovery rates were calculated for each analyte based on target samples that were fortified at three concentration levels (5, 15, and 30 µg·kg^−1^ dw for the analytes EFX and CFX and 1, 5, and 15 µg·kg^−1^ dw for the analytes OTC, 4-epi-OTC, and SCP). Analytes showed recovery rates ranging between 93.0 and 110.5% (Table 4).

The precision of the methodology was assessed by analyzing the repeatability and the intralaboratory reproducibility. The CV for intralaboratory reproducibility was lower than 15% at each level. Meanwhile, the CV for repeatability was lower than that observed for intralaboratory reproducibility at each level (Table 4). In the assessment of intralaboratory reproducibility, the samples were processed on a different day and by a different analyst, inputs from another batch were used, and the brand of glass microfiber paper filters was changed. 

### 3.3. Analysis of Antimicrobial Concentrations in Commercial Lettuce

The application of the analytical method for the detection and quantification of veterinary pharmaceuticals in lettuce obtained from markets showed that 50% of the samples presented detectable concentrations of OTC or EFX. Two samples showed concentrations of OTC + 4-epi-OTC above the LOQ (Table 5).

## 4. Discussion

This work studied AM drugs that were selected due to their critical importance in both veterinary and human medicine [20]. In this context, CFX is deemed a top-priority drug, as are sulfonamides and tetracyclines, which are both classified as highly important by the World Health Organization [33].

The implementation and optimization of the extraction methodology used in this research was based on a method previously published by Albero et al. [12], but it was necessary to add a couple of steps, such as sample filtering and using EDTA buffer as a solvent. These were required because the detection and quantification of AMs in vegetables such as lettuce can be affected by interferents like chlorophyll, vitamins, water, waxy or fatty compounds, and even heavy metals. Thus, preprocessing and cleaning samples becomes paramount to ensure a correct interpretation of the results [34].

Similar to that of other tetracyclines, the nucleus of OTC is a linear tetracyclic structure composed of four fused rings that form chelating complexes with different cations (e.g., Ca^2+^, Mg^2+^, and Fe^3+^) and make the drug insoluble in water [24]. Therefore, using EDTA buffer as a cleaning solvent was essential to take advantage of this multivalent chelating ability of metal cations, improving the extraction of OTC [35]. This scenario of OTC binding metal cations is possible because lettuce does accumulate a large amount of heavy metals within its tissues, mostly in its leaves [36]; using EDTA buffer as a part of this method effectively reduced any interference on the chromatographic analysis that might be attributed to these compounds. The use of EDTA buffer in the extraction of tetracyclines from vegetables has been described by other authors [37,38].

The clean-up step for this methodology involved a dispersive solid phase (d-SPE), that uses C_18_ in bulk instead of solid-phase extraction columns (SPE). The d-SPE technique is advantageous because of several characteristics, such as the reduction in material residues, reduction in the required amount of solvents, optimization of the cleaning time, and considerable reduction in the economic costs of the extraction procedure [39].

Analytical validation establishes experimental evidence that a method consistently delivers results that are reproducible, precise, and accurate when using an established and accepted methodology [40]. As all the parameters evaluated for the purposes of this study met the requirements established by Decision 808/EC of the European Union and VICH GL2: Validation of analytical procedures: methodology [29,30], it is clear that the lettuce methodology developed for this research is effectively capable of precisely and accurately detecting and quantifying the target AM drugs. Moreover, the analysis of commercial lettuce samples confirmed the suitability of this method. 

In this work, we found that nine samples of commercial lettuce showed a signal that was consistent with the presence of residues of OTC + 4-epi-OTC. Seven of these samples showed concentrations below 1 µg·kg^−1^ dw, and two presented concentrations ranging between 1 and 16 µg·kg^−1^ dw. For EFX and CFX, we detected these compounds in three samples at concentrations below 5 µg·kg^−1^ dw, whereas for SCP, no samples were identified that presented detectable residues of this AM. This is the first study to evaluate the presence of antimicrobial residues in lettuce in our country. Therefore, these results are the first approximation of the presence of AM residues in lettuce plants from main Chilean markets. The concentrations detected in this study were similar to those detected by Tadić et al. [41] in the peri-urban area of Barcelona, where they detected concentrations in lettuce of EFX, trimethoprim, and sulfamethoxazole that ranged from 1.9 to 17.3 µg·kg^−1^ dw. 

The presence of AM residues in vegetables has been of great concern. In this context, several studies have estimated the risk from eating lettuce, or other vegetables, with residues of tetracyclines, quinolones, or sulfonamides [42,43,44,45]. In these studies, the concentrations ranged from 0.1 to 27.2 µg·kg^−1^ of dry matter, and the researchers indicated that the risk to human health is insignificant, mainly because the factors involved are many (e.g., concentration of AMs in the vegetable, estimated vegetable consumption, and acceptable daily intake of the residue, among others). However, the researchers emphasized that the presence of AMs in vegetables should not be overlooked, because they are a direct route of exposure to humans.

The low AM drug residues that we detected in commercial lettuce samples could be a hazard to public health, as this product is consumed raw. Additionally, it is already well established by the scientific evidence that, when AMs are present in sub-inhibitory concentrations, they can function as signaling molecules. These signals might result in triggering changes in the expression and transference of bacterial genes, as well as in altering bacterial virulence, causing the formation of biofilms, and accelerating the horizontal transference of genes coding for drug resistance between non-pathogenic bacteria and pathogens, even between phylogenetically distant bacteria [2,46,47,48]. Therefore, it is essential to continue developing further research on this topic.

Currently, different methodologies for AM detection in lettuce have been published [13,19,26,27]; however, none of them involve the detection of both the parental molecules and their main active metabolites or epimers. The advantage of our methodology is that it allows for the detection of more molecules with AM activity, in addition to detecting AM residues in different lettuce varieties, especially when bearing in mind that lettuce is one of the most valued vegetables by Chilean consumers, as evidenced by the more than 8000 hectares dedicated to their cultivation in the year 2022 [49]. Considering this context, the development of an analytical methodology for the detection of AM drug residues in lettuce becomes the foundation for future studies that may explore the transference of AM drugs from soil to the plant tissues of popular vegetables that are consumed fresh, such as is the case for lettuce.

## 5. Conclusions

In this work, we proposed an analytical methodology developed to detect OTC, 4-epi-OTC, EFX, CPX, and SCP in lettuce samples. The method is precise, specific, and linear. The confirmatory nature of this analytical methodology makes it a reliable tool to detect and quantify these analytes. 

This method allowed for the detection of trace residues of oxytetracycline and enrofloxacin residues in commercial lettuce from the main markets of Santiago, Chile. This evidence represents a first approximation to the current food safety status for the lettuce grown in our country. This background information paves the way for future research that may inform possible regulations or help in building surveillance systems against AM drugs in foods of plant origin.

## Figures and Tables

**Table 1:** Elution gradient for HPLC mobile phases.

Time (min)	Phase A (%)	Phase B (%)
0	85	15
5	85	15
5.1	60	40
10	60	40
10.1	10	90
15	10	90
16	85	15
25	85	15

**Table 2:** Masses of the precursor and fragmented quantifier ions of each analyte.

Analyte	Precursor Ion (Da)	Fragmented Ion (Da)	Time (ms)	DP (v)	EP (v)	CE (v)	CXP (v)
Oxytetracycline	461.0 461.0	426.0 ** 381.0 ***	100.0 100.0	72.0 73.0	10.0 10.0	28.0 36.0	25.0 22.0
4-Epi-oxytetracycline	461.0 461.0	426.0 ** 444.0 ***	100.0 100.0	72.0 70.0	10.0 10.0	28.0 30.0	25.0 15.0
Enrofloxacin	361.0 361.0	317.0 ** 343.0 ***	100.0 100.0	50.0 50.0	7.00 7.00	28.0 23.0	20.0 8.0
Ciprofloxacin	332.0 332.0	231.0 ** 314.0 ***	100.0 100.0	56.0 56.0	4.5 4.5	47.0 28.0	4.0 4.0
Sulfachloropyridazine	285.2 285.2	156.0 ** 108.0 ***	100.0 100.0	61.0 61.0	10.0 10.0	21.0 31.0	12.0 8.0
Sulfamethazine 13C6 *	285.3	124.1	100.0	71.0	10.0	31.0	12.0
Enrofloxacin-D5 *	365.1	321.0	100.0	50.0	7.0	23.0	8.0
Tetracycline-D6 *	451.0	160.0	100.0	34.0	10.0	25.0	30.0

Da: dalton; DP: declustering potential; EP: entrance potential; CE: collision energy; CXP: collision cell exit potential; v: volt; *: internal standard; **: quantifier ion; ***: confirmatory ion.

**Table 3:** Linearity of calibration curves for each analyte.

Analyte	R^2^*Average	CV (%)	Slope Average	CV (%)
Oxytetracycline	0.996	0.095	0.966	9.51
4-Epi-oxytetracycline	0.995	0.166	1.111	6.794
Enrofloxacin	0.995	0.38	0.04	14.928
Ciprofloxacin	0.998	0.11	0.33	17.693
Sulfachloropyridazine	0.993	0.257	0.04	3.011

R^2^*: coefficient of determination; CV: coefficient of variation.

**Table 4:** Repeatability, intralaboratory reproducibility, and recovery for each analyte.

Analyte	Working Concentration (µg·kg^−1^ dw)	CV (%) Repeatability	CV (%) Reproducibility	Average Recovery (%)
Oxytetracycline	1	6.25	13.87	93.0
	5	1.99	8.01	99.1
	15	0.18	7.05	97.0
4-Epi-oxytetracycline	1	7.69	11.64	99.8
	5	3.16	3.25	100.1
	15	0.27	0.31	100.0
Enrofloxacin	5	9.74	8.65	110.5
	15	5.09	5.64	94.2
	30	1.04	1.05	101.2
Ciprofloxacin	5	11.61	13.02	101.6
	15	6.13	7.41	99.1
	30	1.25	1.47	100.2
Sulfachloropyridazine	1	6.62	14.02	102.1
	5	0.82	4.03	99.4
	15	0.09	0.38	100.1

dw: dry weight; CV: coefficient of variation.

**Table 5:** Antimicrobial residues in lettuce samples from markets.

Sample ID	OTC + 4-epi-OTC (µg·kg^−1^ dw)	EFX + CFX (µg·kg^−1^ dw)	SCP (µg·kg^−1^ dw)	Lettuce Variety	Market
1	<LOD *	<LOD	<LOD	Butter 1	Lo Valledor
2	Traces **	<LOD	<LOD	Butter 2	
3	<LOD	<LOD	<LOD	Butter 3	
4	<LOD	<LOD	<LOD	Butter 4	
5	<LOD	<LOD	<LOD	Romaine 1	
6	Traces	<LOD	<LOD	Romaine 2	
7	Traces	<LOD	<LOD	Romaine 3	
8	16.004	<LOD	<LOD	Romaine 4	
9	<LOD	<LOD	<LOD	Iceberg 1	
10	<LOD	<LOD	<LOD	Iceberg 2	
11	Traces	<LOD	<LOD	Iceberg 3	
12	Traces	<LOD	<LOD	Iceberg 4	
13	<LOD	Traces	<LOD	Butter 1	Vega Central
14	<LOD	Traces	<LOD	Butter 2	
15	1.384	<LOD	<LOD	Butter 3	
16	Traces	<LOD	<LOD	Butter 4	
17	<LOD	Traces	<LOD	Romaine 1	
18	<LOD	<LOD	<LOD	Romaine 2	
19	<LOD	<LOD	<LOD	Romaine 3	
20	<LOD	<LOD	<LOD	Romaine 4	
21	<LOD	<LOD	<LOD	Iceberg 1	
22	<LOD	<LOD	<LOD	Iceberg 2	
23	Traces	<LOD	<LOD	Iceberg 3	
24	<LOD	<LOD	<LOD	Iceberg 4	

dw: dry weight; * <LOD: lower than the limit of detection; ** Traces: below the limit of quantification (1 µg·kg^−1^ dw). Butter lettuce (*Lactusa sativa var. Capitata*), Romaine (*Lactuca sativa L. var. Longifolia*), and Iceberg (*Lactuca sativa L. var. Crispa*).

## Data Availability

Data is contained within the article.
